# Repeated administration of the noradrenergic neurotoxin *N-*(2-chloroethyl)-*N*-ethyl-2-bromobenzylamine (DSP-4) modulates neuroinflammation and amyloid plaque load in mice bearing amyloid precursor protein and presenilin-1 mutant transgenes

**DOI:** 10.1186/1742-2094-4-8

**Published:** 2007-02-26

**Authors:** Perdita L Pugh, Martin P Vidgeon-Hart, Tracey Ashmeade, Ainsley A Culbert, Zoe Seymour, Marion J Perren, Flora Joyce, Simon T Bate, Anna Babin, David J Virley, Jill C Richardson, Neil Upton, David Sunter

**Affiliations:** 1 Neurology & Gastrointestinal Centre of Excellence for Drug Discovery, GlaxoSmithKline Research & Development Limited, New Frontiers Science Park, Third Avenue, Harlow, Essex, UK; 2 Statistical Sciences, GlaxoSmithKline Research & Development Limited, New Frontiers Science Park, Third Avenue, Harlow, Essex, UK

## Abstract

**Background:**

Data indicates anti-oxidant, anti-inflammatory and pro-cognitive properties of noradrenaline and analyses of post-mortem brain of Alzheimer's disease (AD) patients reveal major neuronal loss in the noradrenergic locus coeruleus (LC), the main source of CNS noradrenaline (NA). The LC has projections to brain regions vulnerable to amyloid deposition and lack of LC derived NA could play a role in the progression of neuroinflammation in AD. Previous studies reveal that intraperitoneal (IP) injection of the noradrenergic neurotoxin *N-*(2-chloroethyl)-*N*-ethyl-2-bromobenzylamine (DSP-4) can modulate neuroinflammation in amyloid over-expressing mice and in one study, DSP-4 exacerbated existing neurodegeneration.

**Methods:**

TASTPM mice over-express human APP and beta amyloid protein and show age related cognitive decline and neuroinflammation. In the present studies, 5 month old C57/BL6 and TASTPM mice were injected once monthly for 6 months with a low dose of DSP-4 (5 mg kg^-1^) or vehicle. At 8 and 11 months of age, mice were tested for cognitive ability and brains were examined for amyloid load and neuroinflammation.

**Results:**

At 8 months of age there was no difference in LC tyrosine hydroxylase (TH) across all groups and cortical NA levels of TASTPM/DSP-4, WT/Vehicle and WT/DSP-4 were similar. NA levels were lowest in TASTPM/Vehicle. Messenger ribonucleic acid (mRNA) for various inflammatory markers were significantly increased in TASTPM/Vehicle compared with WT/Vehicle and by 8 months of age DSP-4 treatment modified this by reducing the levels of some of these markers in TASTPM. TASTPM/Vehicle showed increased astrocytosis and a significantly larger area of cortical amyloid plaque compared with TASTPM/DSP-4. However, by 11 months, NA levels were lowest in TASTPM/DSP-4 and there was a significant reduction in LC TH of TASTPM/DSP-4 only. Both TASTPM groups had comparable levels of amyloid, microglial activation and astrocytosis and mRNA for inflammatory markers was similar except for interleukin-1 beta which was increased by DSP-4. TASTPM mice were cognitively impaired at 8 and 11 months but DSP-4 did not modify this.

**Conclusion:**

These data reveal that a low dose of DSP-4 can have varied effects on the modulation of amyloid plaque deposition and neuroinflammation in TASTPM mice dependent on the duration of dosing.

## Background

Alzheimer's disease (AD) is a chronic debilitating disorder involving impairments in memory function [1], behavioural disturbances [2], neuroinflammation [3,4], synaptic failure [1] and a gradual loss of neurones within the brain [5]. A recent analysis of post-mortem AD brain found that neuronal loss was most severe in the locus coeruleus (LC) rather than in the nucleus basalis, with LC loss correlating best with the duration of illness [6]. The noradrenergic (NA) neurones of the LC project widely throughout the brain, in particular to innervate areas of the cortex and hippocampus [7]. These brain areas, critical to attention and memory processes, are also known to degenerate in AD [5,8]. NA is involved in attention and memory [9-12] and has antioxidant [13,14] and anti-inflammatory[15-17] properties in vitro and in vivo.

Acute intraperitoneal (IP) administration of low-doses (50 μg kg^-1^) of the selective noradrenergic neurotoxin *N-*(2-chloroethyl)-*N*-ethyl-2-bromobenzylamine (DSP-4), in rat, potentiates the expression of pro-inflammatory genes in response to beta amyloid protein (Aβ) injection into the brain [15]. Low-dose DSP-4 administration (50 μg kg^-1^or 5 mg kg^-1^) to transgenic human amyloid precursor protein (APP) mice exacerbated microglial activation and inflammatory gene expression [18], modulated amyloid load [19] and influenced cell survival [20]. Higher doses of DSP-4 (two injections of 50 mg kg^-1 ^spaced by a week,) have been assessed in APP23 mice, resulting in an exacerbation of AD relevant readouts at 6 months post-injection [21]. These data suggest that NA release in the projection areas may underlie a protective mechanism, as well as an involvement in cognitive processes. Drugs that increase brain NA levels, such as α2 adrenoceptor antagonists, provide neuroprotection [22] and improve memory [23,24]. Compromising the NA system appears to render brain tissue more susceptible to the pro-inflammatory effects of Aβ protein [15,16]. As the LC NA system is compromised in AD [6], it is possible that this down-regulation of brain NA can contribute to the progression of disease.

The present studies examined the consequences of NA perturbation by repeated IP injection of a relatively low-dose (5 mg kg^-1^) of DSP-4 to male TASTPM mice. These mice mimic various hallmarks of AD such as high levels of circulating Aβ protein and its deposition in the form of plaques, cognitive and behavioural deficits [25] and neuroinflammation. Unlike recent work [19] in which DSP-4 was injected twice a month, in the present studies DSP-4 was injected once monthly. As depletion of NA may exacerbate some of the features of AD, these studies aimed to modify the readouts of the TASTPM model and importantly to assess any effects on neurodegeneration, which is normally absent in this mouse model of AD.

## Methods

### Animals and treatments

Heterozygote double mutant TASTPM mice were generated at GlaxoSmithKline as previously described [25]. These animals over express the hAPP695swe mutation and the pre-senilin-1 M146V mutation resulting in over production of human APP and beta amyloid protein. These animals show age related cognitive decline and neuroinflammation and have previously been described in detail [25]. All experimental mice were housed singly with free access to Global Rodent Maintained Diet (Harlan Teklad, UK) and water and were maintained in an ambient temperature of 21 ± 1°C, under a controlled light-dark photoperiod (12:12 h) with lights on at 07:00 h.

At study commencement, male TASTPM mice aged 5 months (in-house supply, n = 48) and age matched C57/Bl6J controls (Harlan, UK, n = 48) were assigned to one of four groups as follows: C57BL6 treated with vehicle (WT/VEH), C57BL6 treated with DSP-4 (WT/DSP-4), TASTPM treated with vehicle (TG/VEH) and TASTPM treated with DSP-4 (TG/DSP-4). DSP-4 was purchased from Sigma-Aldrich, UK. At study commencement mice each received an intraperitoneal (IP) injection of either 5 mg kg^-1 ^of DSP-4 or 0.9% sodium chloride vehicle in a dose volume of 1 ml kg^-1^. Thereafter, each mouse was dosed once a month with their respective treatment. Two weeks following their last injection, half of the mice of each treatment group (at 8 months) and later the remaining half (at 11 months) were assessed in the fear conditioning (FC) paradigm (see below for details of assay) and one week later the mice were culled. At both time points, all brains were removed and cut in two along the midline. Half of each brain was post-fixed in 4% paraformaldehyde solution whilst the remaining half was microdisected with cortex and hippocampii removed, frozen on dry ice and stored at -80°C. All experimental procedures were conducted in accordance with GlaxoSmithKline local ethics committee and conform to the UK Animals (Scientific Procedures) Act 1986.

### Ex-vivo neurochemistry-high performance liquid chromatography (HPLC)

Selected tissue samples underwent standard preparation and processing through high performance liquid chromatography – electrochemical detection (HPLC-ECD) for separation analysis against a noradrenaline (NA) standard.

### Tissue preparation

Striatal samples were weighed and homogenised in buffer which comprised 0.4 M perchloric acid containing sodium metabisulphate (0.1% wv^-1^), EDTA (ethylene diamine tetra acetate 0.01% wv^-1^) and L-cysteine (0.1% wv^-1^) at a ratio of 100 μl homogenising buffer per mg of striatal tissue (giving a tissue concentration of 0.01 g/ml) – all constituents of assay buffer were sourced from Sigma, UK. The samples were then centrifuged on 10,000 × g at 4°C for 10 minutes. Supernatant was subsequently fraction decanted.

### HPLC-ECD analysis

Aliquots (30 μl) of supernatant were transferred into micro-volume glass vials for HPLC-ECD analysis. Mobile phase consisted of 0.07 M KH_2_PO_4_, containing 1.5 mM sodium octylsulphonate and 0.1 mM EDTA.Na_2_, MeOH, tetrahydrofuran (87.5:12:0.5%, wv^-1^). Flow rates for optimal separation and detection varied between 2.2 to 2.5 ml/min. Sample aliquots of 10 μl each were automatically injected onto the columns. Separation was performed using two Chromolith Performance columns connected in series (100 × 4.6 mm i.d., Lutterworth UK). Eluates were detected using a Decade electrochemical detector fitted with a glassy carbon cell (Antec, Leyden, The Netherlands) set at + 0.65 V versus *in situ *Ag/AgCl reference electrode. Data were collected using Empower software (Waters, Milford, MA). The chromatograms were compared with internally run noradrenaline standard calibrations (concentrations between 1 and 100 ng/ml) to identify and quantify components. Results were expressed in ng mg^-1 ^of wet-weight tissue and presented as mean ± sem. Effects were analysed by Fischer LSD test.

### Fear conditioning

Contextual fear-conditioning is a form of associative learning, in which animals learn to recognise a training environment (conditioned stimulus) previously paired with an aversive stimulus (foot-shock, unconditioned stimulus). Mice were subjected to the contextual fear conditioning paradigm using a computer controlled fear conditioning system (TSE, Bad Homburg, Germany). On day 1 (training day), mice were placed individually in a grey Perspex shock chamber (25 × 28 × 16.5 cm; Width × Depth × Height) with a metal grid floor, housed within a sound attenuating box. For the conditioning trial, after 180 s habituation to the chamber, the mice received an auditory tone cue for 30 s followed by an electric foot-shock (0.7 mA constant current for 2 s) delivered via the grid floor of the chamber and this tone-shock pairing was repeated after 30 s. Contextual memory was tested 24 h after the conditioning trial. On day 2, the mice were placed in the familiar chamber without tone stimulation or foot-shock and the amount of inactivity was measured in a 180 s trial period. The mice were returned to their home cage whilst a novel chamber with clear Perspex walls, a triangular floor area (using a diagonal box divider) and a grid floor covered with a thick paper mat was prepared. The mice were then placed in the modified chamber and the amount of inactivity in a 180 s trial period was recorded in this novel context. The test sequence of mice was randomised across genotype, drug treatment and chamber and individual chambers were swabbed with 70% alcohol between each trial. Locomotor activity and movement at the same location (e.g. rearing) were monitored by infra-red motility sensors in the chamber walls. Inactivity was defined as movement < 1 cm/s. The percentage duration of inactivity was calculated by the fear conditioning system. On day 2, contextual memory, defined as the amount of inactivity displayed in the familiar context (in which the foot-shocks were received) minus the amount of inactivity displayed in the novel context was measured.

### Immunohistochemistry

Hemisected brains were immersed fixed in 4% paraformaldehyde (VWR, UK) for 3 days at room temperature and prepared for paraffin wax processing using a Shandon Citadel 1000 tissue processor and embedded in paraffin wax using a Shandon Histocentre II embedding centre (Thermo Shandon, UK). Using a Microm HM 355 S rotary microtome (Microm, UK), at least 40 semi-serial sections of 5 μM thickness were prepared from each sample to include the LC and dried at room temperature for at least 24 hours prior to staining. Sections were taken from the same region throughout the LC and 2 sections each were assigned for immunohistochemical staining for tyrosine hydroxylase (TH), glial fibrillary acid protein (GFAP), microglia (CD68) and amyloid immunohistochemistry. Sections were dewaxed in Histoclear (National Diagnostics, UK) and hydrated through industrial methylated spirit (IMS) (VWR, UK), 70% IMS and deionised water. Sections assigned for TH were microwaved (Sanyo Showerwave, 1000 W) in tris-borate-EDTA buffer, pH 8.3 (Sigma-Aldrich, UK) for 2.5 minutes at 1000 W then for a further 10 minutes at 450 W then allowed to cool for 20 minutes. Sections were washed in deionised water and a hydrophobic barrier applied above and below the section using a PAP pen (DakoCytomation, UK). Slides were loaded into an automated immunostaining machine (DakoCytomation, UK) and Optimax buffer (A. Menarini, UK) was applied to each section. Sections received peroxidase block (DakoCytomation, UK) for 5 minutes, primary antibody rabbit anti TH, affinity purified (Chemicon International, UK) diluted 1 in 500 in antibody diluent (DakoCytomation, UK) for 30 minutes, biotinylated goat anti rabbit (Vector Laboratories, UK) diluted 1 in 200 in Optimax buffer for 30 minutes, peroxidase ABC kit (Vector Laboratories, UK) for 45 minutes and diaminobenzidine substrate kit (DakoCytomation, UK) for 10 minutes with Optimax buffer wash between each step and deionised water after the diaminobenzidine step. Slides were taken out of the machine and washed in running tap water for 5 minutes prior to staining in Gills haematoxylin (HD Supplies, UK) for 3 seconds. Sections were washed in running tap water to "blue", dehydrated in graded followed by absolute IMS, cleared in Histoclear and mounted in DPX (VWR, UK). For CD68 (for activated microglia) and glial fibrillary acidic protein (GFAP) for reactive astrocytes, sections were dewaxed and hydrated as before and treated with proteinase K (DakoCytomation, UK) for 5 minutes followed by deionised water wash prior to loading onto the immunostaining machine. Endogenous peroxidase activity was blocked with peroxidase for 5 minutes and either rat anti mouse CD68 (Serotec, UK) dilution 1 in 50 in antibody diluent for 30 minutes or rabbit anti bovine GFAP 1 in 500 in antibody diluent was applied for 30 minutes. For CD68 the secondary antibody was biontinylated anti rat (Serotec, UK) dilution 1/100, for GFAP biotinylated anti rabbit dilution 1 in 200 in Optimax buffer for 30 minutes at room temperature. Sections were treated with peroxidase ABC for 45 minutes at room temperature and DAB substrate for 10 minutes. For amyloid immunohistochemistry we used in house monoclonal antibody 1E8, raised against the 13–27 fragment of Aβ. Sections were dewaxed and hydrated as before then treated with 85% formic acid (VWR, UK) for 8 minutes then washed thoroughly in deionised water before applying the hydrophobic barrier and loading onto the immunostaining machine. The machine was programmed to deliver the ready to use peroxidase block (DakoCytomation, UK) for 5 minutes, primary mouse monoclonal antibody 1E8 diluted 1 in 1000 in antibody diluent (DakoCytomation, UK) for 30 minutes, prediluted labelled streptavidin biotin system (LSAB) 1 (DakoCytomation, UK) for 10 minutes, prediluted LSAB 2 for 10 minutes and diaminobenzidine substrate kit (DakoCytomation, UK) for 10 minutes with Optimax buffer wash between each step and deionised water after the diaminobenzidine step. Slides were taken out of the machine and washed in running tap water for 5 minutes prior to staining in Gills haematoxylin (HD Supplies, UK) for 3 seconds. Sections were washed in running tap water to "blue", dehydrated in graded then absolute IMS, cleared in Histoclear and mounted in DPX (VWR, UK).

For TH counting, the cell bodies in the LC were viewed under a ×10 objective on an Olympus BX41 microscope. Photomicrographs of 1E8 stained sections, for amyloid, were taken using a Colourview digital camera and AnanlySIS image analysis software (Soft Imaging Systems) and ×4 objective, a percentage area measurement was calculated using Leica Q-Win system. GFAP and CD68 sections were assessed but not quantified.

### TaqMan analyses

Total RNA was isolated from cortex tissues from WT and TASTPM mice using Trizol reagent according to the manufacturer's instructions. The RNA was resuspended in ultraPURE distilled water (Invitrogen, life technologies, UK), and RNA purity was confirmed by ensuring that A260:A280 nm ratio was >1.8. Equal quantities of RNA from each tissue sample were used in reverse transcription reactions to generate cDNAs. First strand cDNA syntheses and aliquoting of resulting cDNA products for subsequent parallel Taqman PCR reactions were all performed as described in detail previously [26]. Additional reactions were performed using genomic DNA to produce a standard curve relating threshold cycle to template copy number. Primer (F and R) and probe (P) sets were designed from mouse or rat sequences in the Genbank database using Primer Express software (Perkin-Elmer, UK); see Table 1 for sequences. All Taqman probes contained 6-Carboxyfluorescein at 5' end and the quencher dye, 6-carboxy-tetramethyl-rhodamine at the 3' end.

**Table 1:** TaqMan reagent sequences

**Gene**	**Reagent Sequences**
**MIP-1α**	F; AGCTGACACCCCGACTGC
	R; GTCAACGATGAATTGGCGTG
	P; TGCTGCTTCTCCTACAGCCGGAAGAT
**MIP-1β**	F; AGCTCTGCGTGTCTGCCC T
	R; GCTGAGAACCCTAGAGCACA
	P; TCTCCT CTTGCTCGTGGCTGCCTT
**TNFα**	F; TCCAGGCGGTGCCTATGT
	R; GAGCGTGGTGGCCCC
	P; TCAGCCTCTTCTCATTCCTGCTTGTGG
**iNOS**	F; TGATGTCCGAAGCAAACATCA
	R; TGTGGCTCCCATGTTGCAT
	P; TTCAGATCCCGAAACGCTTCACTTCC
**IL-1β**	F; TTGGGCCTCAAAGGAAAGAAT
	R; TCTCCAGCTGCAGGGTGG
	P; TATACCTGTCCTGTGTAATGAAAGACGGCA CA
**IκBα**	F; CGGAGGACGGAGGACTCGTT
	R; ACTTCCATGGTCAGCGGCT
	P; TGCACTTGGCAATCATCCACGAAGA
**FasL**	F; TACCACCGCCATCACAACC
	R; TTTGTGTTGTGGTCCTTCTTCTTTAG
	P; CTCCCACTGCCGCCACTGACC
**GFAP**	F; GGAGCTCAATGACCGCTTTG
	R; AGCGCCTTGTTTTGCTGCTC
	P; CAGCTACATCGAGAAGGTTCG
**RANTES**	F; TCTTGCAGTCGTGTTTGTCAC
	R; TCTTGAACCCACTTCTTCTCT
	P; AGGAACCGCCAAGTGTGTGC
**CCR5**	F; GGAATGACACACTGCTGCCTAA
	R; GAACACTGAGAGATAACTCCGGAAC
	P; CCCTGTCATCTATGCCTTTGTTGGAGAGA

## Results

### HPLC analyses

**Figure 1:**
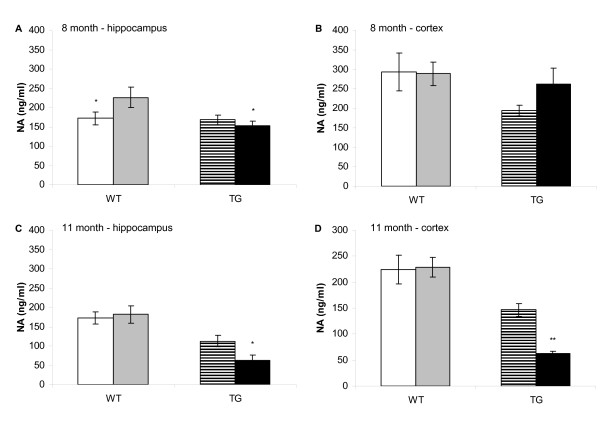
**Noradrenaline levels in hippocampus and cortex**. For all graphs WT/VEH (white), WT/DSP-4 (grey), TG/VEH (stripes), TG/DSP-4 (black). NA levels in hippocampus (A) and cortex (B) in WT and TG mice (8 months). Hippocampal NA levels are significantly lower in WT/VEH (p < 0.05) and TG/DSP-4 (p < 0.05) mice relative to WT/DSP-4 mice. In cortex TG/VEH mice show a trend towards lower NA levels compared with WT/VEH (p = 0.08) and WT/DSP-4 (p = 0.09). TG/DSP-4 NA levels are no different to WT/VEH or WT/DSP-4. (Mean ± S.E.M. ng/ml; WT/VEH 293 ± 49; TG/VEH 194 ± 14; WT/DSP-4 289 ± 30; TG/DSP-4 262 ± 41). At 11 months, NA levels are significantly reduced in both hippocampus (p < 0.05) (C) and cortex (p < 0.05) (D) in TG mice (hippocampus: mean ± S.E.M. ng/ml, WT/VEH 172 ± 16; TG/VEH 113 ± 15; cortex: WT/VEH 224 ± 28; TG/VEH 147 ± 12). TG/DSP-4 mice have lower levels of cortical (p < 0.0001) and hippocampal (p < 0.0001) NA compared with WT/DSP-4 (cortex: mean ± S.E.M. ng/ml, WT/DSP-4 229 ± 19; TG/DSP-4 63 ± 5; hippocampus: WT/DSP-4 182 ± 23; TG/DSP-4 62 ± 14). TG/DSP-4 mice have lower levels of cortical (p < 0.01) and hippocampal (p < 0.05) NA compared with TG/VEH.

At 8 months of age (figure 1a) the level of NA in hippocampus or cortex of WT mice was not altered by DSP-4 treatment compared with vehicle treatment. There was a trend towards a reduction in cortical NA levels in TG/VEH mice compared with WT/VEH mice at 8 months of age (p = 0.08), indicating a reduced (33%) basal cortical NA level in TASTPM mice compared with WT mice. Levels of NA in TG/DSP-4 mice were no different to the levels seen in WT/VEH (p = 0.6) or WT/DSP-4 (p = 0.6).

By 11 months of age (figure 1b), DSP-4 treatment did not alter the levels of NA in hippocampus or cortex of WT mice when compared with WT/VEH. Basal levels of NA in hippocampus (34% reduction, p < 0.05) and cortex (34% reduction, p < 0.05) were significantly lower in the TG/VEH mice compared with WT/VEH and significantly lower in hippocampus (65% reduction) and cortex (72% reduction) of TG/DSP-4 mice compared with WT/DSP-4 (p < 0.0001, for both brain areas). DSP-4 treatment of TASTPM mice, however, did significantly further reduce hippocampal (45% reduction, p = 0.049) and cortical (57% reduction, p = 0.005) NA levels compared with TG/VEH. This effect of DSP-4 to reduce NA, apparent only in the TASTPM mice, suggests a raised susceptibility to the deleterious effects of this toxin in the TASTPM mice at the dose concentration and dosing regime that was used in the present study. However, this effect to reduce NA levels in TASTPM was only observed at the 11 month time point, the time point when a significant reduction in the level of TH staining in the LC of TG/DSP-4 mice is observed (see below).

### Fear conditioning

#### Contextual fear association

**Figure 2:**
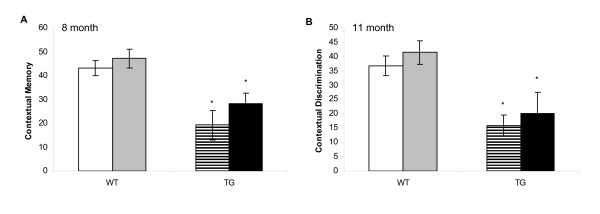
**Contextual discrimination in the fear conditioning paradigm**. Contextual discrimination in the fear conditioning paradigm in WT and TG mice at 8 months (A) and 11 months (B). Compared with respective WT mice, TG mice show impairment in contextual discrimination, a measure of the percent inactivity during 180 seconds during when mice are placed back in the original environment where foot-shock was first administered. DSP-4 treatment did not modify the level of impairment seen in TG mice. *p < 0.05 vs. respective WT group.

Irrespective of treatment, WT mice exhibited robust and similar contextual memory at both time-points investigated whilst TG mice exhibited a similar and significant impairment of contextual memory compared with the WT group. Following either 3 or 6 month treatment, DSP-4 had no effect on contextual memory in either genetic group; both WT and TG groups treated with DSP-4 displayed similar contextual memory to that in the appropriate vehicle-treated group (figure 2).

### Immunohistochemistry

**Figure 3:**
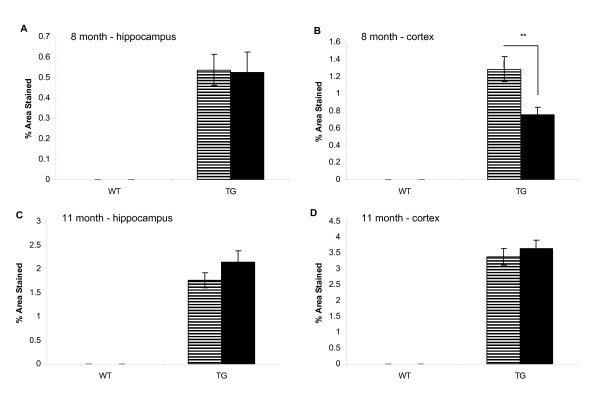
**Amyloid histology**. Percentage area of stain for diffuse amyloid at 8 and 11 months in hippocampus (A,C) and cortex (B,D). There is a reduction in cortical amyloid in TG/DSP-4 vs. TG/VEH at 8 months, **p < 0.01.

**Figure 4:**
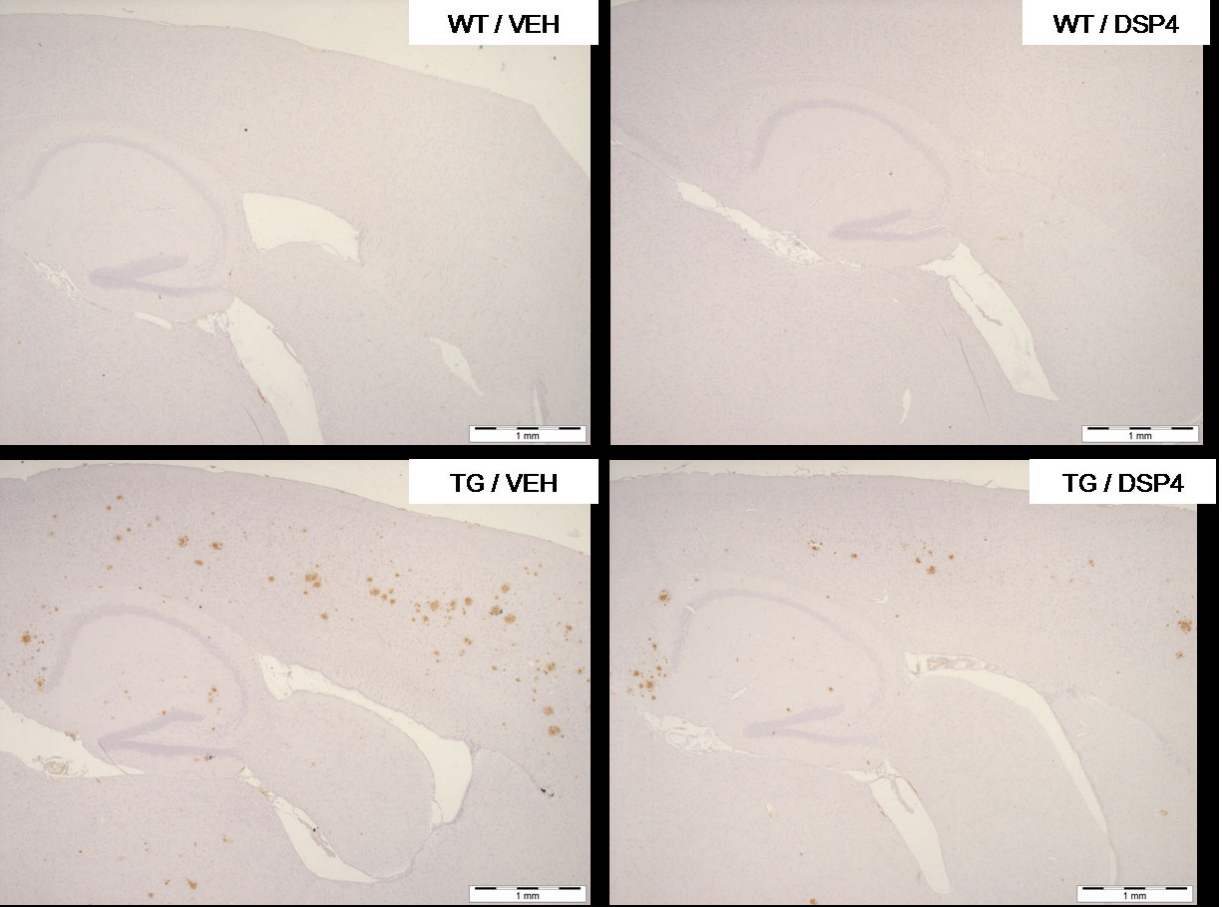
**Amyloid histology – representative sections (8 months)**. Sagittal sections across cortex and hippocampus for amyloid staining (8 months) in WT and TG mice. Note the marked reduction in cortical amyloid staining in TG/DSP-4 group compared with TG/VEH. Scale bar represents 1 mm.

**Figure 5:**
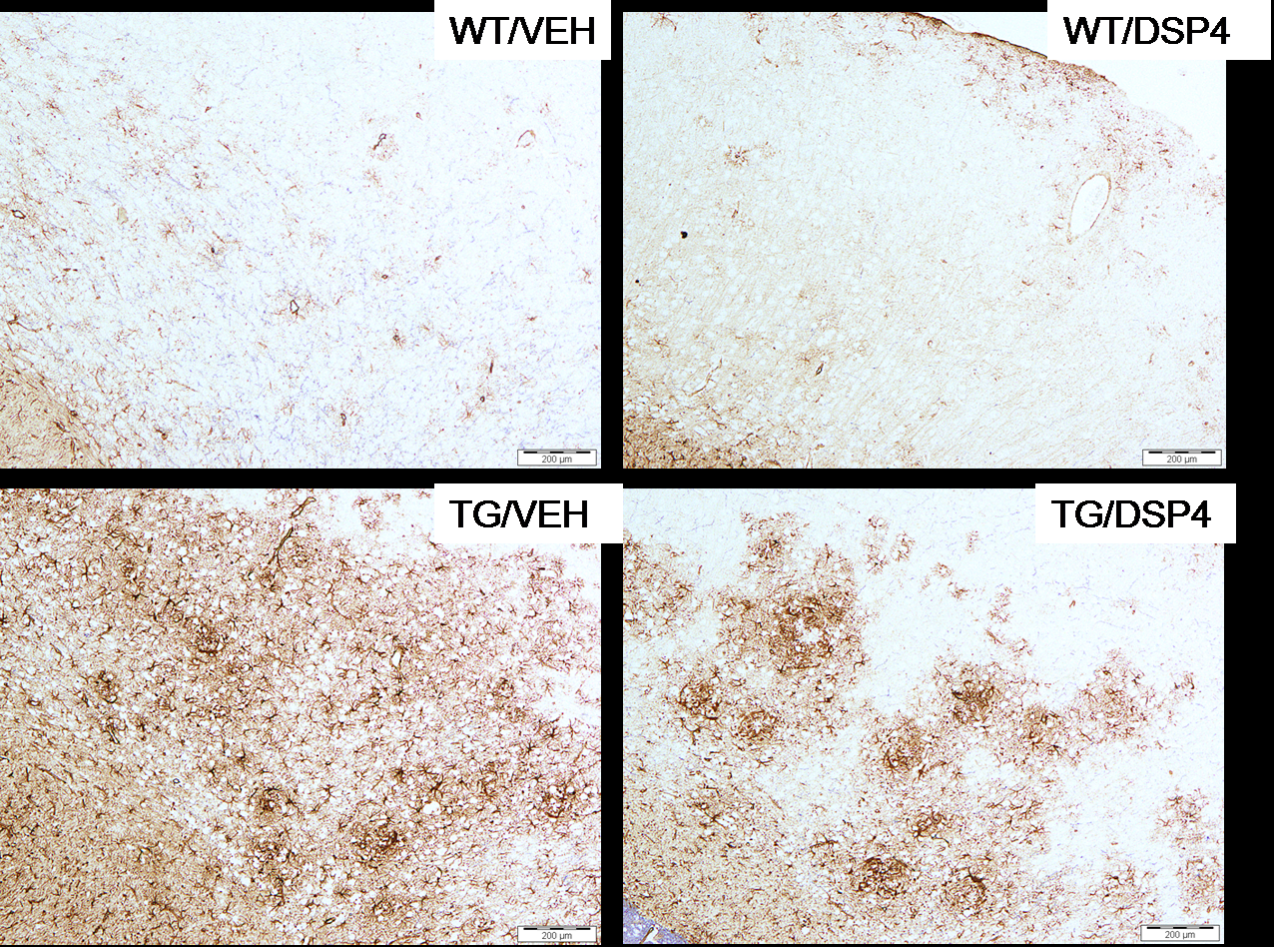
**Astrocytic staining (8 months).** Astrocytic staining (8 months) in WT and TG mice. At 8 months staining is more patchy and less uniform in the TG/DSP-4 compared with TG/VEH group. Scale bar represents 200 μm.

**Figure 6:**
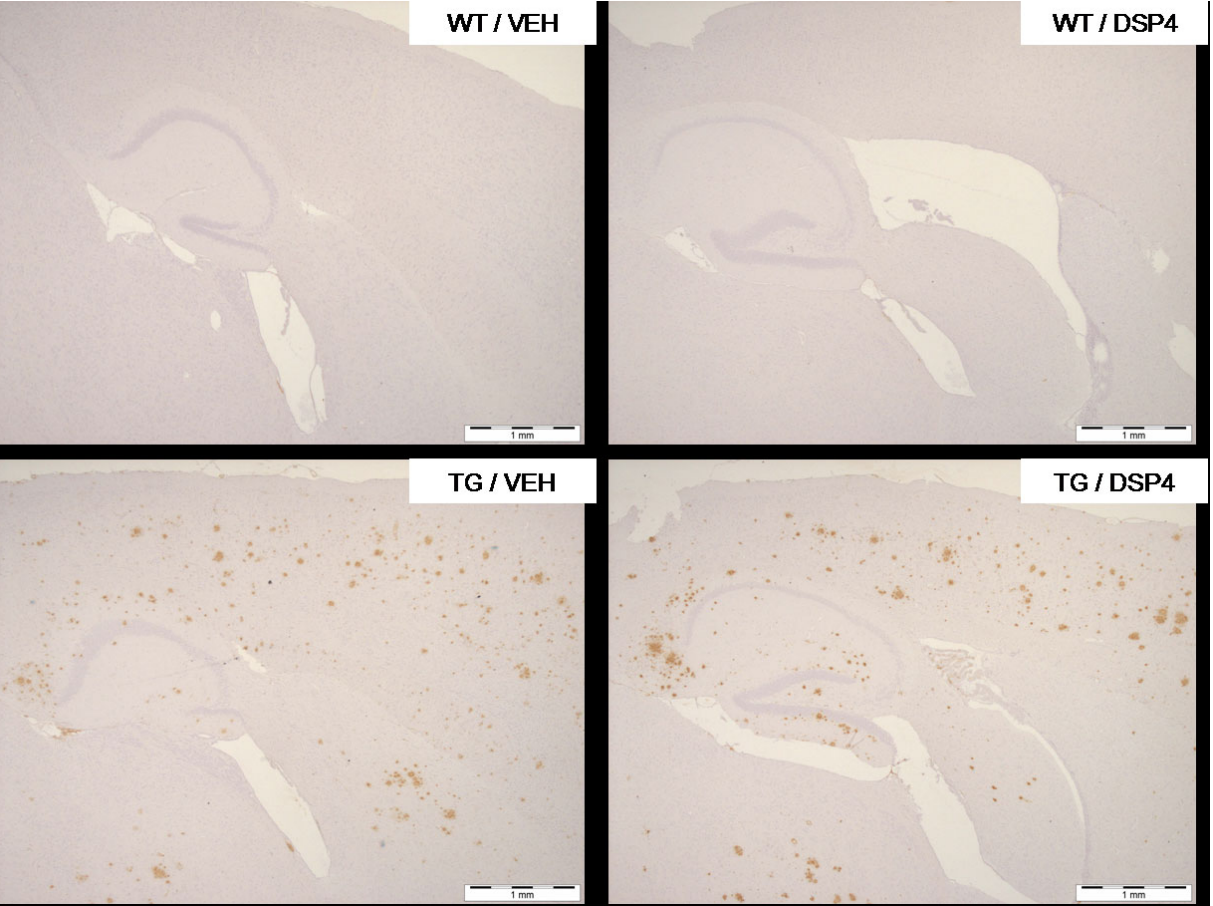
**Amyloid histology – representative sections (11 months).** Sagittal sections across cortex and hippocampus for amyloid staining (11 months) in WT and TG mice. At 11 months staining is of a similar level in TG/DSP-4 and TG/VEH groups. Scale bar represents 1 mm.

**Figure 7:**
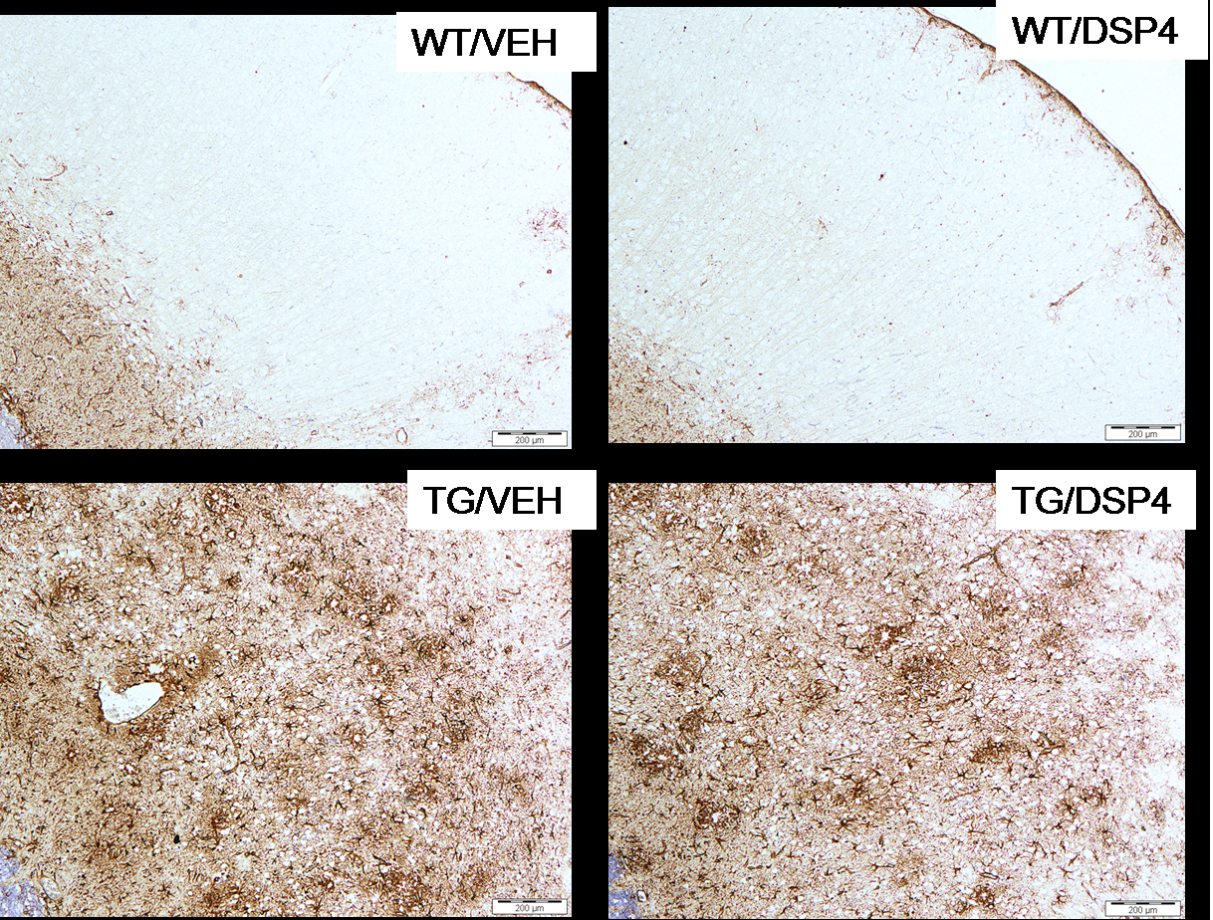
**Astrocytic staining (11 months)**. Astrocytic staining (11 months) in WT and TG mice. At 11 months the staining pattern is similar in TG/DSP-4 and TG/VEH groups. Scale bar represents 200 μm.

**Figure 8:**
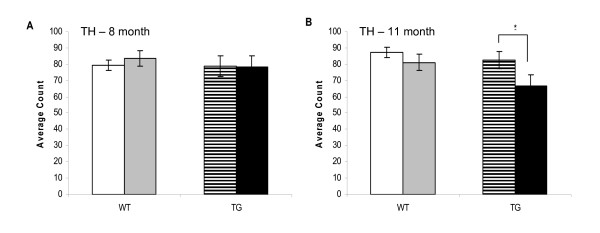
**Tyrosine hydroxylase histology in locus coeruleus**. LC TH positive cell counts at 8 months (A) and 11 months (B) in WT and TG mice. There is a significant reduction in cells staining positive for TH in TG/DSP-4 vs TG/VEH at 11 months. *p < 0.05.

Brains were processed for a number of markers including diffuse amyloid plaques, activated microglia, reactive astrocytes and tyrosine hydroxylase (TH). At 8 months of age, there was a significant reduction in the area of cortex stained positive for diffuse amyloid plaques in TG/DSP-4 mice compared with TG/VEH (figure 3B, figure 4). There was a non-significant difference in the area of diffuse amyloid plaque in hippocampus of TG/DSP-4 mice compared with TG/VEH at this time point (figure 3A, figure 4). Although there was no difference in the area of microglial stain in cortex between the two TASTPM groups at 8 months, the cortical GFAP stain for reactive astrocytes was patchy and less dense in the TG/DSP-4 mice compared with TG/VEH (figure 5). This was supported at the transcriptional level by a reduction in GFAP mRNA measured in the cortex of TG/DSP-4 compared with TG/VEH at 8 months. By 11 months of age both TG/DSP-4 mice and TG/VEH mice had comparable levels of diffuse amyloid plaque, microglial (data not shown) and astrocytic staining in the areas examined (figures 3C, 3D, 6 and 7). Although there was no difference in the levels of LC TH expression across all groups at 8 months (figure 8A), histological examination of the LC at 11 months revealed a significant reduction in TH levels in the LC of TG/DSP-4 mice compared with TG/VEH (figure 8B). Levels of TH in LC of TG/VEH mice were no different to WT mice of either treatment.

### TaqMan analyses

**Figure 9:**
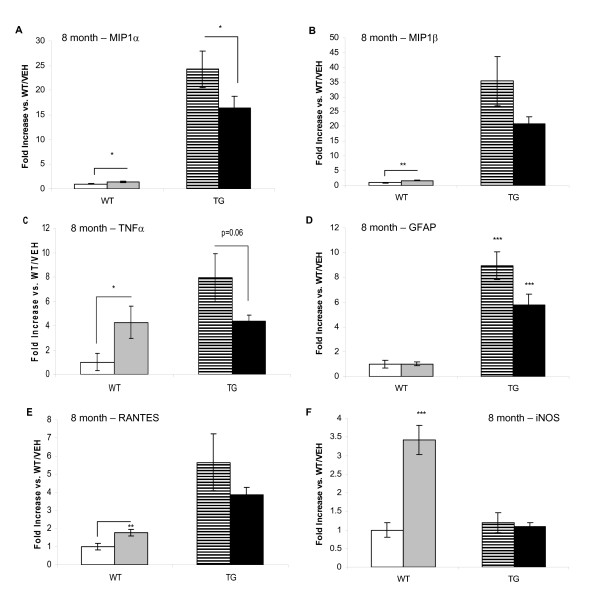
**mRNA levels in cortex, 8 months**. mRNA levels in WT and TG mice (8 months). mRNA levels are raised in TG/VEH vs. WT/VEH for MIP1-α (A), MIP1-β (B), TNF-α (C), GFAP (D) RANTES (E). There is a significant reduction in MIP1-α mRNA levels in TG/DSP-4 vs. TG/VEH. DSP-4 increased iNOS (F) in WT mice only and iNOS was not raised in TG/VEH or TG/DSP-4 vs. WT/VEH.

Hemisected cortex was analysed by TaqMan PCR for various mRNA's that encode mediators involved in inflammation. Macrophage inflammatory protein-1alpha (MIP-1α), macrophage inflammatory protein-1beta (MIP-1β), tumour necrosis factor-alpha (TNF-α), GFAP and 'Regulated upon Activation, Normal T cell Expressed and Secreted' chemokine (RANTES) mRNA levels were significantly increased in TG/VEH compared with WT/VEH group at 8 months (figure 9). However, there was a trend towards a reduction in the levels of some of these mRNA in the TG/DSP-4 mice compared with TG/VEH mice. In the case of MIP-1α, TG/DSP-4 mice showed a statistically significant reduction in MIP-1α mRNA compared with TG/VEH. Although levels were reduced, the level of MIP-1β mRNA in TG/DSP-4 was not statistically different to levels in TG/VEH (p = 0.12). There was also a trend towards a reduction in TNF-α (p = 0.06) and GFAP (p = 0.15) mRNA levels in TG/DSP-4 compared with TG/VEH. In WT mice, DSP-4 treatment increased RANTES and inducible nitric oxide synthase (iNOS) mRNA levels compared with vehicle treatment but DSP-4 treatment did not further alter the levels of these mRNA in TG mice.

**Figure 10:**
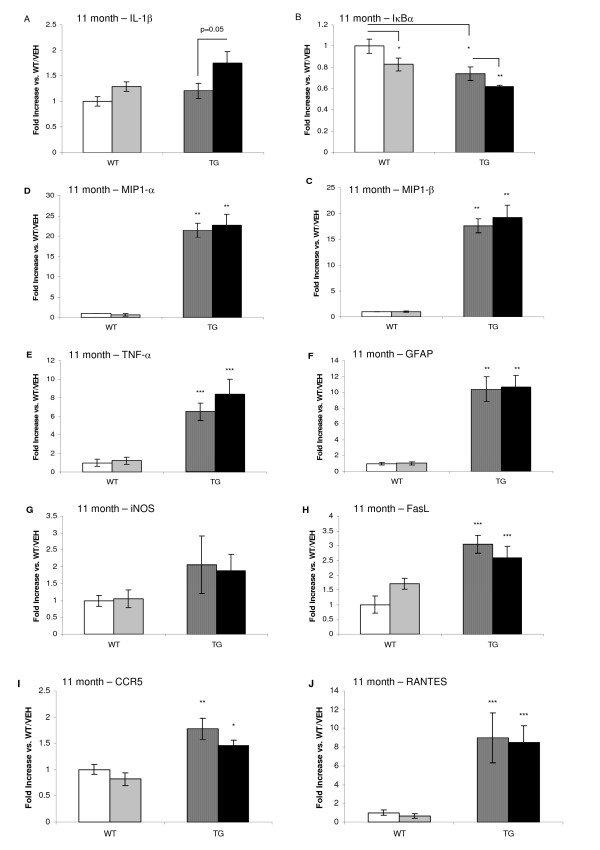
**mRNA levels in cortex, 11 months**. mRNA levels in WT and TG mice (11 months). mRNA levels are raised in TG/DSP-4 vs. TG/VEH for IL-1β (A). Levels of IκBα are reduced in TG mice and DSP-4 treatment reduces IκBα in both WT and TG mice (B). TG mice have elevated MIP1-α (C) and MIP1-β (D) TNF-α (E), GFAP (F), FasL (H), CCR5 (I), RANTES (J) are raised in TG mice and DSP-4 does not modify this. ***p < 0.001, **0.01, *0.05 vs. WT/VEH and DSP-4 treatment does not modify this.

By 11 months of age, DSP-4 treatment of WT mice did not cause an increase in interleukin-1 beta (IL-1β) levels compared with vehicle treated WT mice. However, IL-1β levels were increased in TG/DSP-4 mice compared with TG/VEH (p = 0.05) (figure 10). This indicates that the DSP-4 exacerbated the inflammatory profile for IL-1β only in the TASTPM mice. DSP-4 lowered mRNA levels for the nuclear factor kappa B (NFκB) inhibitory subunit, IκBα, in WT mice. In vehicle and DSP-4 treated TASTPM mice, IκBα mRNA levels were significantly lower than those levels measured in WT/VEH mice. TG/DSP-4 mice displayed the lowest levels of IκBα mRNA and this level was significantly different to TG/VEH levels. A reduction in IκBα leads to increased NFκB expression. NFκB regulates the expression of inflammatory cytokines, chemokines, immunoreceptors and cell adhesion molecules and as such is a key mediator of the immune response. MIP-1α, MIP-1β, TNF-α and GFAP levels were all significantly increased in TASTPM mice compared with WT at 11 months (figure 10). The pro-apoptotic Fas ligand (FasL), RANTES and chemokine co-receptor-5 (CCR5 – which binds MIP-1α, MIP-1β and RANTES) mRNA were also significantly elevated in TASTPM compared with WT (figure 10) and DSP-4 treatment did not modify the levels of these markers in TASTPM mice at this 11 month time point.

## Discussion

These studies highlight the importance of the NA system in the modulation of readouts relevant to AD in the TASTPM mouse. The readouts we examined included NA levels and LC TH, Aβ protein deposition, neuroinflammation and behaviour following monthly intraperitoneal DSP-4 administration for either 3 or 6 months duration. In general, previous studies have employed doses of DSP-4 which are ten-fold the dose used in the present studies, which may explain why such studies show a loss in TH in LC in both TG and WT mice [21]. Our studies used the more subtle dose of 5 mg kg^-1 ^with which we did not see overt changes in LC TH immunoreactivity or changes in brain NA levels in WT mice. However, we did see a significant reduction in LC TH immunoreactivity and large reductions in NA in the brain of TASTPM mice which had been treated with DSP-4. We can observe from the HPLC analyses that there is a non-significant trend for a decline in brain NA levels of TG/VEH compared with WT/VEH mice at 8 months and a significant reduction by 11 months. Hence, there is a natural decline in brain NA levels in TG/VEH controls, suggesting that the NA system is already perturbed in TASTPM mice and could explain why they are more reactive to the effects of the low dose of DSP-4. At the 8 month time point, there is a tendency for increased cortical NA in TG/DSP-4 relative to the TG/VEH group that is not evident in the hippocampus. Based on the data generated, it is difficult to explain why these different brain areas show differing sensitivity to DSP-4. A time-course study is required in order to track the changes in NA levels throughout the 6 months of DSP-4 treatment. Our sample represents a snap-shot and as such does not provide information on the fluctuations in brain NA occurring over time following DSP-4 treatment. The decrease in LC TH immunoreactivity seen in TG/DSP-4 mice is small in contrast to other literature [21], but this can be explained by the lower dose of DSP-4 and differing protocol of administration employed in the present studies. In addition, the LC TH decrease seen in the present studies is small when compared with the LC cell loss that can be seen in AD [6]. The lack of changes in brain NA and in LC TH immunoreactivity in WT mice suggests that the low dose of DSP-4 used is not sufficient to affect mice in which the NA system is not already perturbed.

Previous data reports that modulation of the LC NA system impacts on the regulation of inflammatory mediators in vitro and in vivo. In vitro studies have shown an inhibition of microglial inflammatory responses (nitric oxide and interleukin-1β production) by NA [27]. Anti-inflammatory actions of α2 adrenoceptor antagonists, which act to increase extracellular NA by inhibiting the pre-synaptic inhibition by NA on its own release, have also been reported in vivo [28]. Conversely, it has been reported that NA depletion in vivo, by injection of DSP-4, leads to an exacerbation of neuroinflammation in response to central injection of Aβ protein [15,19]. The anti-inflammatory action of NA is mediated through the modulation of chemokine [29] and cytokine [30] release from macrophages and microglia. In vitro studies of microglia have shown that NA, α1 agonists, β1 and β2 agonists can each suppress the expression of mRNAs encoding the pro-inflammatory cytokines, IL-6 and TNF-α [30]. NA has also been shown to suppress the microglial release of nitric oxide (NO) [30,31]. Recently, NA was reported to reduce the microglial induced cell death of cortical neurones, an effect shown to be mediated via reduction of IL-β release from microglia [32].

In the current studies, histological analyses revealed a patchy GFAP positive astrocytic stain in cortex of TG/DSP-4 group compared with a more diffuse pattern seen in TG/VEH. This histological readout of a reduced inflammatory reaction was backed up by TaqMan PCR analyses of mRNA. As well as a decrease of GFAP protein by histology at 8 months there was also a reduction in GFAP mRNA and a reduction in mRNA for other proteins involved in inflammation including MIP-1α, MIP-1β and TNF-α. Interestingly, despite a non-significant difference in NA levels between vehicle and DSP-4 treated WT groups, DSP-4 treatment significantly increased iNOS, TNF-α and RANTES mRNA expression in WT mice at the 8 month time point, an effect which was not observed in TASTPM mice. Based on the current data we cannot confirm the reason for this discrepancy and further studies to fully assess the effects of DSP-4 on brain mRNA expression and how this relates to NA levels should be performed. Our current data only provides information on NA levels at the time of sampling. Further investigations will require a more in-depth analysis involving multiple measurements taken following DSP-4 treatment.

The present data report that once monthly injection of 5 mg kg^-1 ^DSP-4 to TASTPM mice is anti-inflammatory and slows down amyloid plaque accumulation, as seen from 5 through to 8 months of age. However, by 11 months of age, the profile is similar across both DSP-4 treated TASTPM groups. In order to interpret these data, in particular the observed beneficial effects of DSP-4 in TASTPM mice, it is necessary to consider the actions of DSP-4 in vivo. Although high doses (50 mg kg^-1^) of DSP-4 have been shown to dramatically reduce tissue NA levels and ultimately lead to a cell loss in the LC of normal mice, the effects of lower doses on brain NA levels have not been reported in detail. Interestingly, it has been reported by microdialysis that 50 mg kg^-1 ^of DSP-4 acutely *increases *the extracellular concentration of NA in the frontal cortex in normal rats [33,34]. In our current studies, in the short term (3 months of once monthly dosing from 5–8 months of age), the 5 mg kg^-1 ^dose of DSP-4 did not decrease tissue NA levels in TASTPM mice when compared with vehicle treated TASTPM mice. We show that our low dose of DSP-4 acutely prevented a natural decline in extracellular NA in cortex in the short term, as was seen in TG/VEH mice. At this 8 month time point there was no change in LC TH cell count, indicating an intact NA system. TG/VEH mice, however, did show a trend towards reduced cortical NA which was associated with an increased inflammatory profile and increased amyloid load compared with TG/DSP-4. This suggests that the trend of increased NA in the TG/DSP-4 group may have slowed inflammatory processes and the amyloid accumulation that is usually present by this time point, as evidenced by the TG/VEH readout.

Kalinin et al recently demonstrated that depletion of NA in the brains of V717F APP over expressing mice treated with DSP-4 corresponded with an increase in the number of amyloid plaques [19]. In the current study, decreased cortical NA levels in TG/VEH relative to TG/DSP-4 mice related to increased amyloid in the cortex of TG/VEH mice at the 8 month time point. TG/VEH and TG/DSP-4 mice showed similar hippocampal NA levels at the 8 month time point which may explain why no difference in hippocampal amyloid load was observed between the groups. By 11 months, despite a large reduction in cortical and hippocampal NA levels and a small but significant decrease in LC TH staining in TG/DSP-4 mice, amyloid plaque levels were similar to the TG/VEH group. Extending such a study beyond an 11 month time point may reveal if DSP-4 causes greater amyloid plaque load relative to vehicle treated TASTPM mice. Our data and previously reported literature illustrate that NA can modulate amyloid plaque deposition; however, reported data is contradictory as to the actions of noradrenergic agonism on amyloid plaque load. Kalinin et al (2006) reported that NA can increase the microglial phagocytosis of amyloid plaques in vitro whilst, in vivo, DSP-4 treatment decreased the expression of the amyloid degrading enzyme neprilysin [19]. In contrast, the administration of a beta2-adrenergic agonist, which would mimic the effects of NA, resulted in the augmentation of cerebral amyloid plaque deposition in APP/PS1 over expressing mice [35]. Hence, how and in what way NA impacts on the regulation of amyloid plaques in vivo remains an issue of debate.

Various brain areas, including the hippocampus, cortex and amygdala, are thought to be involved in the FC response [37]. The hippocampus and cortex are innervated by the LC NA system [36] and NA can modulate beta amyloid load. Hence, we reasoned that noradrenergic depletion of these areas via the administration of DSP-4 may influence performance in FC. DSP-4 treated TASTPM mice, although showing a reduced plaque load in cortex, displayed a similar level of FC impairment relative to vehicle treated TASTPM at 8 months of age. Hippocampal amyloid load was not affected by DSP-4 and hence we cannot discount that FC performance may have improved if DSP-4 had also reduced plaque load in this area. At 11 months of age the impairment in FC was again of an equal magnitude in both TASTPM groups and the deficit in TG mice was not exacerbated by DSP-4, although by this stage amyloid levels were indistinguishable between groups. By 11 months there was also no further worsening of the FC impairment compared with 8 months in the TG/VEH group or the WT/VEH group. Hence, either the mice were maximally impaired in the FC assay by 8 months or the cause of the FC deficit is amyloid independent. Further characterisation of the onset of the FC deficit with relation to brain amyloid and the tracking of any further decline in FC will be important in addressing this issue. The relative contributions of the hippocampus, amygdala and cortex to contextual learning remain debated. In the current studies, we cannot account for any impact the LC NA system may have had on the amygdala as this area was not examined for amyloid load or NA levels.

The various studies to date which report DSP-4 administration to APP or APP/PS1 mice all differ with respect to a number of parameters which include the DSP-4 dose used, dosing regime (single vs. repeated injections), transgenic model used and the age of the mice at the initiation and end of dosing. This makes direct comparison across studies difficult. It is possible that in our studies, at even later time-points or with twice monthly as opposed to once monthly DSP-4 injection, the inflammatory profile and amyloid plaque load in TG/DSP-4 mice would be greater than that seen in TG/VEH mice. The recent data [19] certainly provide strong evidence that twice-monthly dosing of 5 mg kg^-1 ^DSP-4 to mutant V717F human amyloid precursor protein (APP) mice over a 6-month period caused a marked increase in inflammation and amyloid plaque load in brain when measured after 6 months. It is of importance to observe earlier readouts in the mutant V717F APP mouse using the twice monthly DSP-4 paradigm reported by Kalinin et al [19] to see if these mice also show an earlier effect on inflammation and amyloid similar to that seen in our studies.

## Conclusion

In conclusion, our data add novel information with respect to the effects of DSP-4 on NA perturbation, amyloid load, neuroinflammation and LC cell survival in a transgenic mouse model bearing APP and PS1 mutant transgenes. Future work will address the effects of the boosting of central NA in TASTPM mice, for example with α2 adrenoceptor antagonists, to assess the impact this may have on amyloid plaques, neuroinflammation and behaviour. It is envisaged that such an approach will be beneficial for the treatment of AD.

## Competing interests

The author(s) declare that they have no competing interests.

## Authors' contributions

PLP carried out all in vivo work and was involved in experimental design and interpretation of data. MPV-H carried out histological analyses, developed new protocols and provided quantification. TA carried out HPLC measurements and developed protocols. AC was involved in study design and the TaqMan analyses. ZS carried out TaqMan analyses. MP carried out all the fear conditioning work. FJ carried out histological analyses and interpretation of data. STB performed all statistical analyses. AB carried out HPLC measurements. DV was involved in experimental design. JR created the TASTPM mice and was involved in study design. NU provided critical assessment of data. DS co-wrote the manuscript, led the series of studies and co-conducted all in vivo work. All authors read and approved the final manuscript.
